# Ultrasound for hip dysplasia – disparities between community and tertiary paediatric services

**DOI:** 10.1111/ans.70000

**Published:** 2025-02-14

**Authors:** Ye Zhu, Melody Feng, Ishith Seth, Rekha Ganeshalingam, Monsurul Hoq, Leo T. Donnan

**Affiliations:** ^1^ Royal Melbourne Hospital Murdoch Children's Research Institute Flemington Road Parkville Victoria Australia; ^2^ Murdoch Children's Research Institute Monash University; ^3^ Department of Orthopaedics University Hospital Geelong, Murdoch Children's Research Institute; ^4^ Department of Clinical Epidemiology and Biostatistics Royal Children's Hospital, Murdoch Children's Research Institute; ^5^ Department of Orthopaedics Royal Children's Hospital, Murdoch Children's Research Institute

**Keywords:** developmental dysplasia of the hip, orthopaedics, paediatrics, radiology, ultrasonography

## Abstract

**Objective:**

Explore the differences in ultrasound reporting between community ultrasound centres and a high‐volume paediatric tertiary centre in diagnosing developmental dysplasia of the hip (DDH).

**Participants:**

One hundred infants less than 6 months of age from a paediatric hip registry, who underwent ultrasounds in both community and tertiary centres within a 2‐week interval.

**Results:**

One percent of a 200‐hip sample reported scan quality. Alpha angles were reported in 84.5% (*n* = 169) of community reports and 78.5% (*n* = 157) of tertiary centre reports. Femoral head coverage was reported similarly in both environments at 94.5% in community and 93.5% in tertiary. Beta angle appeared in 45% (*n* = 90) and 2% (*n* = 4), respectively. Bland Altman plots revealed that there existed variation in ultrasound interpretation especially in more dysplastic hips. The concordance correlation coefficient showed only moderate agreement (Rho = 0.60 for alpha angles, Rho = 0.61 for femoral head coverage), and interrater reliability on hip classification reached moderate agreement (Cohen's Kappa = 0.57 for alpha angle, and 0.45 for femoral coverage) between community and tertiary hospital reports.

**Conclusions:**

Reliance on the community‐acquired ultrasound report as a true reflection of the state of a hip is not completely justified. The lack of standardized reporting poses challenges for community clinicians in starting treatment or making appropriate referrals. Only a moderate agreement has been observed between the community and tertiary scans. Consequently, over 25% of hips classified as abnormal are potentially normal, and more than 15% dysplastic hips could be undetected.

## Graphical abstract

Ultrasonography is crucial for assessing hip dysplasia in infants less than 6 months. Community clinicians rely on ultrasound reports rather than images for management decisions. However, the absence of standardized guidelines for reporting leads to varied practices. Our study found that although there is moderate agreement between the community and tertiary scans, greater degrees of dysplasia resulted in increased variance in reporting. Consequently, over 25% of hips classified as abnormal are potentially normal, and more that 15% dysplastic hips could be undetected. Future efforts should focus on standardization of reporting, credentialling centres, and improving training for community ultrasonographers.

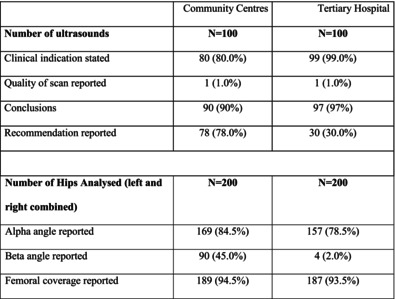

## Introduction

Developmental dysplasia of the hip (DDH) is a paediatric condition characterized by abnormal development of the acetabulum and the femoral head, resulting in mechanical instability within the hip joint.^1^ Ultrasonography (US) serves as the primary imaging modality for evaluating potential dysplasia in the infant's hips before the age of 6 months when risk factors for DDH are present or clinically relevant abnormalities are detected through physical examination.^2^ Ultrasound is a safe and radiation‐free method of assessing the hips offering real‐time imaging and the capability of performing dynamic assessment.^3^, ^4^ It is commonly requested by paediatricians and general practitioners outside of tertiary paediatric centres to assess suspected hips.

Ultrasound examination of the hip is complex, necessitating the acquisition of high‐quality images in the “standard plane”^5^ for geometric and morphological evaluation along with dynamic imaging of the hip for stability.^6^ The reliability of ultrasound is contingent on the expertise of both the technician acquiring the images and the radiologist interpreting and reporting the scans. The geometric values and morphological comments of each hip are crucial components of each report, allowing for documentation and comparison of findings, and guiding decision‐making.^7^, ^8^, ^9^

In the Australian health system, hip ultrasound is conducted in both community‐based imaging centres and tertiary hospitals. However, variation in expertise in hip ultrasound among ultrasonographers and radiologists across different centres have been observed. Currently, there is no accreditation process for ultrasound providers, nor are there standards regarding the minimum number of examinations required to maintain a high level of expertise. The situation is compounded by an absence of universal guidelines for hip ultrasound reporting, and a lack of service standards.^10^, ^11^ It is currently unknown if the quality of ultrasound examination and reporting in the community is of an acceptable standard, or how this standard compares to high‐volume centres with experienced ultrasonographers. This raises questions about repeatability and reliability of ultrasound imaging acquisition and measurement.^12^ These issues are concerning as the quality and reporting of the ultrasound scans greatly influence the decision‐making of general practitioners and paediatricians when managing infants with DDH. It is likely that community clinicians rely on the ultrasound report, rather than self‐evaluation of the images, to direct management and decide on the need for specialist referral, potentially leading to under diagnosis or over treatment of DDH.

This study aims to address the aforementioned questions by investigating the disparity in hip ultrasound reporting between community imaging centres in one state of Australia and a major tertiary specialist referral centre. The goal is to obtain a better understanding on the reliability of the reporting upon which community clinicians rely for making management decisions.

## Methods

### Study design and participants

This study employed a retrospective approach to objectively explore and compare the differences in reporting quality and hip measurements between hip ultrasound studies performed at community‐based radiology centres and to those conducted at a high‐volume tertiary paediatric hospital. A consecutive sample of 100 infants who were referred to the tertiary centre between April 2018 to April 2022 with suspected hip dysplasia on initial ultrasound was identified in the paediatric hip registry. These infants underwent a second ultrasound at the tertiary centre, conducted by experienced ultrasonographers specializing in DDH, within 2 weeks of their initial ultrasound conducted in the community. It was felt that under these conditions the hip ultrasound examination should not have changed to any significance. Participants were excluded from the study if they were over the age of 6 months, had their repeat hip ultrasound at the tertiary centre more than 2 weeks after their initial community ultrasound, or had received treatment between the two ultrasounds.

Three investigators independently evaluated and recorded the ultrasound reports and data, including alpha angles, beta angles, femoral head coverage percentage, morphology, and conclusions, from all hip ultrasound reports and data were entered into the REDCap database.^13^ Subsequently, two senior orthopaedic surgeons verified the data. With the high value placed on conclusions from radiology reports among clinicians, we also analyzed this aspect of the information collected. The data from each ultrasound report was assessed in line with its associated conclusion to confirm if the conclusion was accurate, ambiguous or erroneous. The study protocol was designed in compliance with the Declaration of Helsinki, ensuring adherence to ethical guidelines and principles for research involving human subjects. Ethical approval for the study was obtained from The Royal Children's Hospital Research Ethics Committee. All authors had full access to all of the data included in the study.

### Statistical analysis

Both qualitative and quantitative data analysis were utilized to analyse the reports. All analyses were conducted in Stata 16*. Categorical variables from the hip reports were summarized using number and percent, and continuous variables were summarized using mean and standard deviation. The quality of the ultrasound was explored utilizing the known strong correlation between the alpha angle and percentage cover of the hip on ultrasound.^14^, ^15^ The correlation was assessed by Pearson's Correlation Coefficient (r) and presented graphically using scatter plots.

The agreement between the community and the tertiary report in measuring alpha angles or femoral coverage percentages as continuous variables was assessed using the Concordance correlation coefficient (CCC) and presented graphically using the Bland–Altman plot. A CCC value of 0.8 or higher is generally considered to indicate good agreement between the two variables, 0.6 and 0.8 indicate moderate agreement, and below 0.6 indicates poor agreement.

In addition, alpha angles and coverage percentages were dichotomized to abnormal and normal. An alpha angle of under 60° and coverage of under 50% were classified as abnormal. Sensitivity and specificity of identifying abnormal hips using the community reports in comparison to the tertiary reports were calculated along with agreement and inter‐rate reliability (Cohen's Kappa). A kappa value of 0.41–0.60 was considered moderate agreement, 0.61–0.80 substantial agreement, and 0.81–1.00 almost perfect agreement.^16^

The study did not stratify and analyse the risk factors for DDH among the included infants, as these would have no effect on the ultrasound reporting.

## Results

### Overall report characteristics

Ultrasound reports from over 30 community‐based centres were included in this study. Descriptive statistical analysis on the reports from community‐based centres showed that the average age at the time of scan was 10.9 (±6.5 standard deviation (SD)) weeks. The average time for patients to have a repeat scan at the tertiary paediatric hospital was 9.2 (± 3.7 SD) days.

There was minimal reporting of scan quality in either group (2 reports, 1% in total). There was a high level of reporting of femoral head coverage in both groups (376 reports, 94% in total). The community centres utilized the Graf method more frequently as reflected in the much higher reporting of the beta angle (90 reports, 45%), which is a prerequisite for that method (Table 1).

**Table 1:** Comparison of report basics and geometric measurements

	Community centres	Tertiary hospital
**Number of ultrasounds**	***N* = 100**	***N* = 100**
Clinical indication stated	80 (80.0%)	99 (99.0%)
Quality of scan reported	1 (1.0%)	1 (1.0%)
Conclusions	90 (90%)	97 (97%)
Recommendation reported	78 (78.0%)	30 (30.0%)
**Number of hips analyzed (left and right combined)**	***N* = 200**	***N* = 200**
Alpha angle reported	169 (84.5%)	157 (78.5%)
Beta angle reported	90 (45.0%)	4 (2.0%)
Femoral coverage reported	189 (94.5%)	187 (93.5%)

Conclusions were reported at a high rate in both groups (385 reports, 96.3% in total). Community centres reported Graf classification (70 reports, 35%) more frequently than the tertiary hospital, which none reported Graf (0 report, 0%) (Table 2).

**Table 2:** Qualitative variable reporting and analysis of ambiguous/erroneous conclusions

	Community centres	Tertiary hospital
**Number of hips analyzed (left and right combined)**	***N* = 200**	***N* = 200**
Morphology reported	153 (76.5%)	145 (72.5%)
Graf reported	70 (35.0%)	0 (0.0%)
Conclusion reported	189 (94.5%)	197 (98.5%)
Ambiguous conclusions	8 (4.0%)	6 (3.0%)
Erroneous conclusions	26 (13.0%)	25 (12.5%)
*Inaccurate conclusion*	*23 (88.5%)*	*21 (84.0%)*
*Missed immaturity*	*8 (30.8%)*	*7 (28.0%)*
*Inaccurate use of immaturity*	*6 (23.1%)*	*4 (16.0%)*
*Others miscellaneous*	*4 (15.4%)*	*3 (12.0%)*

Ambiguous and erroneous conclusions were identified in 17% (34 reports) of the community scans and in 15.5% (31 reports) of the tertiary. Erroneous conclusions have been further sub‐classified into four different categories. Notably, certain erroneous conclusions were found to fall into more than one category, resulting in the total percentage exceeding 100% (Table 2).

### Difference in geometric analysis of scans

**Fig. 1:**
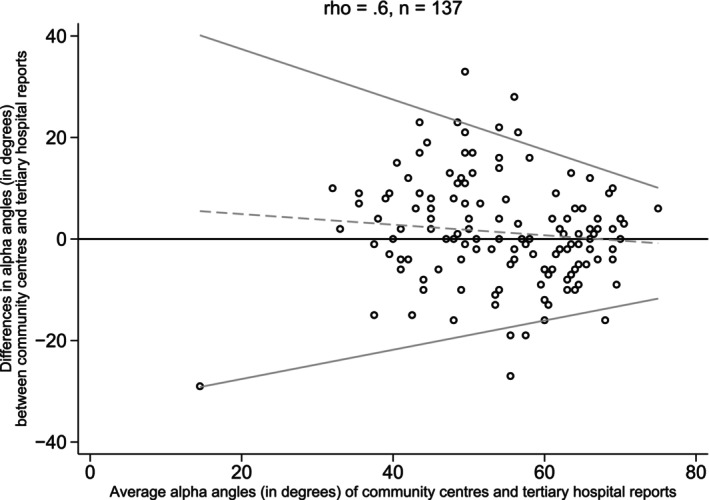
Bland–Altman Plot – Comparison of alpha angle between community and tertiary reports (right and left hips combined).

A total of 137 pairs of alpha angles were compared. The CCC indicated moderate agreement (Rho = 0.6) in alpha angles between community and tertiary reports. The difference between the community and tertiary alpha angles (indicated by the dashed line in Fig. 1) decreased as the average of the two angles approached the normal value of 60°. Additionally, the variability of the difference in alpha angle (the standard deviation) also decreased as the average alpha angle trends towards normal (Fig. 1).

**Fig. 2:**
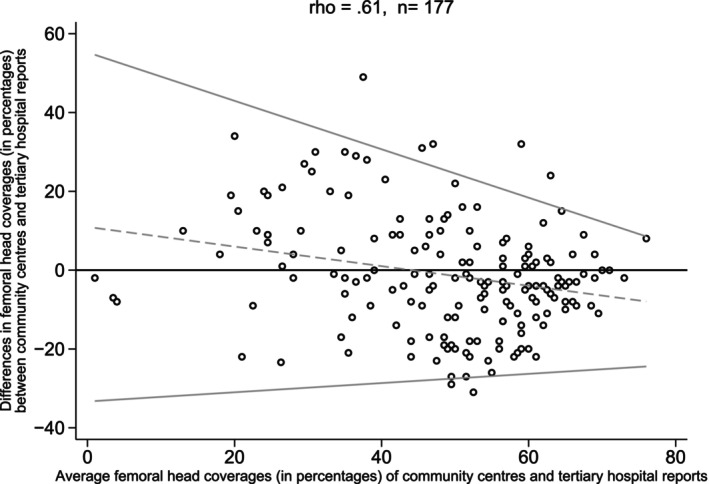
Bland–Altman Plot – Comparison of percentage of femoral cover between community and tertiary reports (right and left hips combined).

A total of 177 pairs of femoral head coverage were compared. The CCC indicated moderate agreement (Rho = 0.61) in femoral head coverage between community and tertiary reports. The difference between the community and tertiary femoral head coverage (indicated by the dashed line in Fig. 2) decreased as the average of the two percentages approached the value of 70%. Additionally, the variability of the difference in femoral head coverage (the standard deviation) decreased as the average alpha angle got closer to normal (Fig. 2).

### Quality of ultrasound

**Fig. 3:**
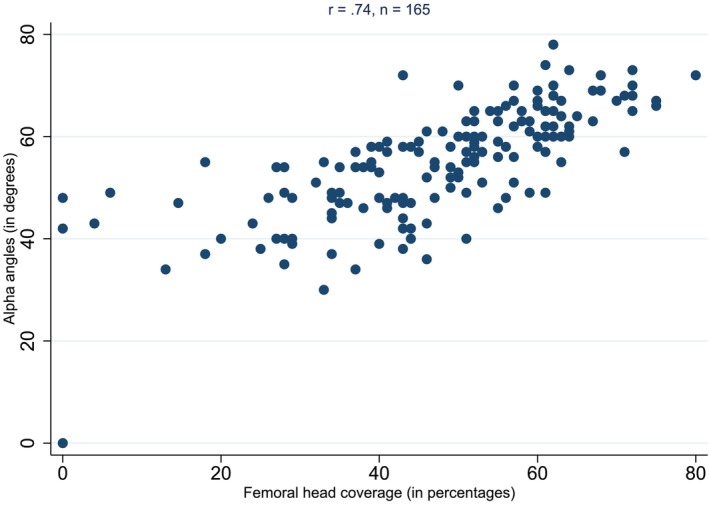
Scatterplot for the Association between alpha angle and percentage of femoral coverage in community reports.

A total of 165 hips from the community reports that reported both alpha and percentage coverage reported are represented in Figure 3. A moderate level of correlation (*r* = 0.74) was observed.

**Fig. 4:**
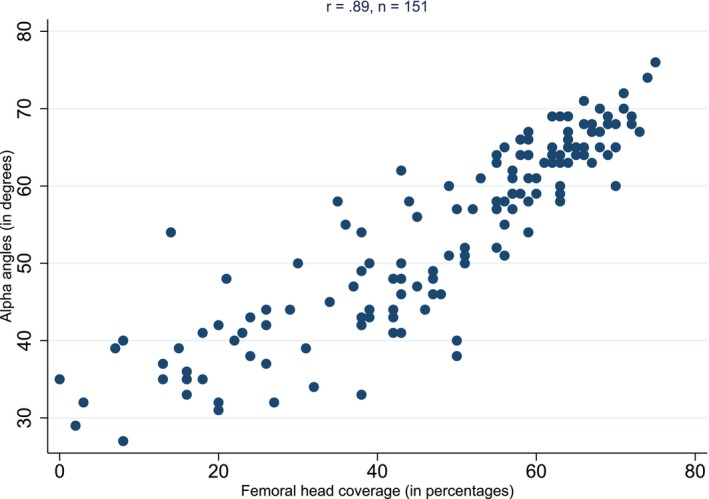
Scatterplot for the association between alpha angle and percentage of femoral coverage in tertiary reports.

A total of 151 hips from the tertiary reports that reported both alpha and percentage coverage reported are included in Figure 4. A very strong level of correlation (*r* = 0.89) was observed.

### Agreement and diagnostic potential of community‐based ultrasound

The community reports were able to identify up to 68 out of 80 abnormal hips (85% sensitivity), and 41 out of 57 normal hips (72% specificity). The agreement between community and tertiary reports in classifying normal and abnormal hips is 79.6% (109/137) based on alpha angles. The interrater reliability indicates moderate agreement (Cohen's Kappa = 0.57, 95% Confidence Intervals 0.43 to 0.72) between community and tertiary reports in classifying hips based on alpha angles (Table 3).

**Table 3:** Diagnostic potential of community‐based ultrasounds based on alpha angle

Alpha angle	Tertiary hospital		
Community centres	Less than 60°	60° or more	Total
Less than 60°	68	16	84
60° or more	12	41	53
Total	80	57	137

The community reports were able to identify 52 out of 70 abnormal hips (74% sensitivity), and 77 out of 107 normal hips (72% specificity). The agreement between community and tertiary reports in identifying normal and abnormal hips is 72.9% (129/177) based on femoral coverage. The interrater reliability indicates moderate agreement (Cohen's Kappa = 0.45, 95% Confidence Intervals 0.32 to 0.59) between community and tertiary reports in classifying hips based on femoral coverage (Table 4).

**Table 4:** Diagnostic potential of community‐based ultrasounds based on femoral coverage

Coverage	Tertiary hospital		
Community centres	Less than 50%	50% or more	Total
Less than 50%	52	30	82
50% or more	18	77	95
Total	70	107	177

## Discussion

Hip ultrasound remains the most important screening tool for infants suspected of having hip dysplasia. Australia has non‐specific guidelines for hip ultrasound practices including that of reporting standards, accreditation, and critical patient volume, leading to very different practices and advice being given.^17^ It had been our observation that community ultrasound reports and recommendations were often at odds to what was being found at repeat scans. Therefore, this contemporary study analyzed and evaluated 100 pairs of hip ultrasound reports from infants under the age of 6 months, taken at different times, from multiple centres between April 2018 and April 2022.

The basics of reporting were similar in both environments except that the tertiary reports had a low rate of recommendations, as it was known that all scans were being reviewed by experienced treating clinicians. It was surprising that there was virtually no mention in either group as to the quality of the scan upon which all measurements are made. This is a worrying finding as any recommendations should be tempered if the scan quality is unknown, as the community clinician relies on the findings and recommendations to decide on next steps in management. This phenomenon raises questions about the repeatability of the study, quality assurance, and performance evaluation in radiology centres.

We were able to infer scan quality by utilizing the previously reported high correlation between alpha angle and percentage cover.^14^, ^15^ Community scans demonstrated a moderate correlation of *r* = 0.74 with the tertiary scans being of a considerably higher quality *r* = 0.89 indirectly indicating a higher quality of imaging. This suggests that the quality of ultrasound examination is more accurate in the tertiary centre.

In evaluating hip dysplasia, the Graf method relies on geometric measures and the assessment of morphology.^5^ The method was used sparingly in the community scans and not at all in the tertiary setting. The alpha angle represents the bony acetabular coverage, and this was not reported in all cases despite being a reliable measure of dysplasia. There appeared to be a greater reliance on percentage cover (376 reports, 94% in total) than any other geometric measure, indicating a move away from the specialized knowledge of the Graf classification of which this measure is not used. We noticed that the application of the Graf method, when used, was reasonable but poor application accounted for most of the ambiguous and inaccurate conclusions (10.5% of conclusions).

As geometric measures are of such importance in the assessment of hip dysplasia, the team specifically looked at how much the community scans varied from the tertiary. Bland–Altman plots for both the alpha angles and femoral head percentage coverages achieved moderate agreement between the two groups, however, the variability was considerably greater in the more dysplastic hip. There are several potential reasons why this may occur. For instance, the level of experience of the sonographer may affect the quality of the scan; greater levels of dysplasia may make obtaining a standard plane view more difficult; and the unsettled infant may be problematic to scan. These three factors alone can result in both over and under‐reporting of dysplasia and could suggest that some community centres may have less experience with infant hip ultrasound compared to the tertiary centre. A greater difference in geometric measurements in the more dysplastic hips plausibly indicate lower accuracy in identification and greater difficulty in diagnosing hip dysplasia in the community.

It is difficult to determine the diagnostic potential of community‐acquired scans compared to that of a tertiary centre using one statistical model. But in the best‐case scenario utilizing thresholds of less than 60°^18^ for alpha angle, ~15% of hips would have been missed and 28% referred as abnormal when they were in fact normal. If a percentage cover of less than 50% is accepted as abnormal,^19^ then 26% would have been missed and 28% referred as abnormal when they were normal. For clinicians who practice in the community and rely wholly on the ultrasound reports this is problematic for both missed diagnosis and overtreatment.

Furthermore, from the study it was determined that a basic ultrasound report should at least contain the following information so that a meaningful and comparative report could be generated: age of the patient, clinical indication, quality of the scan, alpha angle, percentage cover, dynamic scan, conclusion, and recommendation. Specific morphological features (cartilage roof, cartilage rim and bony roof) are not included as they are highly correlated to the geometric measures and has very poor intra and interrater reliability.^11^ In this study, only a little over 50% of reports achieved this level of reporting in either group evaluated. In situations where ultrasound images are not available for review alongside the reports, there is a risk of both diagnostic confusion and misjudging the true condition of the hip.

This study is not without limitations. Only what was reported could be analyzed and this does not consider other information collected that is not reported but utilized to make the report. In a small number of cases the same radiologist working in two different sites reported on both the community and tertiary scans introducing a slight bias in our data. We were not able to determine which community centres were higher volume centres nor perform a sub‐analysis on how quality relates to volume due to the broad number of radiology centres included the study. Future studies would look to perform this analysis and look into opportunities to improve the quality and reliability of hip ultrasound in infants.

## Conclusions

The overall standard of ultrasound reporting for hip dysplasia is highly variable and has the potential to misdiagnose or result in unnecessary treatment. The importance of the quality of investigation as a determinant of accuracy of measurement has been inadequately considered. Surrogate measures utilized in this study have highlighted concerns regarding scan reliability and quality, particularly in the community scans. To ensure an accurate ultrasound assessment of the infant's hip, it is essential to mandate standardized training in image acquisition and interpretation, and that a sufficient volume of scans is maintained to ensure maintenance of experience and skills. It would greatly enhance the interpretation of hip ultrasound reporting to add a statement on quality of the study and any difficulties encountered as a standard part of the report.

There is a clear need for improvements in both the acquisition and reporting of hip ultrasound. Missed hip dysplasia can result in lifelong disability and over‐treatment with bracing is not only burdensome on families but can lead iatrogenic avascular necrosis of the hip.^20^ Community centres provide a valuable and accessible resource for patients and clinicians but may require additional collaboration to optimize the services they provide. Accreditation pathways for sonographers and radiologists along with recommendations for minimal numbers of scans performed for skill maintenance as well as opportunities from the tertiary hospitals to sustain these numbers are some possible solutions. The paediatric radiologist should work hand in hand with the sonographer and be cognisant of scan quality in reporting findings and making recommendations. The reports should be of a standard format so that referrers can be confident that a quality examination has been performed and what next steps need to be taken including that of a repeat scan or specialist referral.

## Author contributions

**Ye Zhu:** Data curation; methodology; writing – original draft; writing – review and editing. **Melody Feng:** Data curation; methodology; writing – original draft; writing – review and editing. **Ishith Seth:** Methodology; writing – original draft. **Rekha Ganeshalingam:** Conceptualization; methodology; supervision; writing – review and editing. **Monsurul Hoq:** Formal analysis; software. **Leo T. Donnan:** Conceptualization; methodology; project administration; supervision; writing – review and editing.

## Conflicts of interest

None declared.
